# Sex and gender bias in major trauma care: a scoping review

**DOI:** 10.1186/s13049-026-01596-3

**Published:** 2026-03-24

**Authors:** A Ghika-Nanchen, L Marzorati, A Merra, C Girardello, P Truong, PN Carron, T Nutbeam, C Clair, FX Ageron

**Affiliations:** 1 Emergency Department, BH 09, Lausanne University Hospital, Bugnon 46, Lausanne, 1011 Switzerland; 2 Emergency Department, Hopital Inter-Cantonale de La Broye, Payerne, Switzerland; 3 University of Plymouth, Plymouth, UK; 4 IMPACT, Centre for Post-Collision Research Innovation and Translation, Devon Air Ambulance, Exeter, UK; 5 Department of Ambulatory Care, Unisanté, University of Lausanne, Lausanne, Switzerland

**Keywords:** Gender, Major trauma, Sex disparity, Sexism, Polytrauma, Emergency medicine

## Abstract

**Background:**

Sex/gender bias have been well-documented in clinical medicine. However, few studies have assessed sex/gender disparities specifically in major trauma care. This scoping review aims to explore sex and gender-based differences in the emergency management of severely injured patients.

**Methods:**

A systematic literature review was conducted in the following electronic databases: Medline Ovid ALL, Embase, CINAHL with Full Text, Web of Science Core Collection, Cochrane Central Register of Controlled Trials with search criteria including keywords and mesh terms: gender, sex, major trauma, wounds and injuries. Three reviewers conducted the article selection.

**Results:**

Seventy-four full-text articles were included in the study. Main themes of sex/gender-based differences were mechanism of injury, severity of trauma, trauma triage, trauma care, mortality, and complications. Women were older with more low-energy trauma than men. Women were more likely to suffer from pelvic and spinal cord injuries. Women were more likely to be under-triaged and under-treated. Sex/gender-based differences in mortality were inconsistent across studies. Adjusted mortality appeared similar between women and men, with conflicting evidence of increased mortality in men in some studies.

**Conclusion:**

Women received less trauma care and were less likely to be transported to a trauma centre. These disparities are not fully explained by differences in injury mechanism or severity and instead appear to reflect modifiable features of trauma systems, particularly triage and transfer practices. Addressing these inequities will require system-level changes to ensure that access to specialist trauma care is based on clinical need rather than sex or gender.

**Supplementary Information:**

The online version contains supplementary material available at 10.1186/s13049-026-01596-3.

## Background

Major differences have been highlighted between women and men both in their health status and medical treatment. These differences could be due to biological and physiological sex differences or could be influenced by gender as a social or cultural construct. In this review, we considered each term according to its specific definition. The term "sex" refers to biological aspects, whereas the term "gender" refers to social aspects. We will use the term sex/gender in our article to reflect its complex intersectional nature, requiring consideration of both biological and social factors as well as their interactions. According to these definitions, the term "gender bias" refers to behaviour that favours one gender over another such as stereotypes or discrimination, while "sex disparity" refers to measurable differences in outcomes based on biological sex [1].

Sex/gender bias has been well studied in cardio-vascular diseases such as acute coronary syndrome and hypertension. Huber et al. showed that women with acute coronary syndrome were less likely to undergo coronary angiography and to receive primary coronary intervention and coronary bypass [2]. In emergency medicine, sex/gender inequities have been highlighted in many areas including diagnosis, pain management and stroke identification [3]. Despite recognition of sex/gender inequity in health, most clinical areas in emergency medicine have not been fully assessed [4]. Indeed, most randomised clinical trials conducted in emergency medicine do not report sex-disaggregated analyses, as shown by Chamberlain et al. [5], with only 1 out of 24 randomised clinical trials including such an analysis. Sex/gender bias therefore represents an important issue in emergency medicine, as the impact of sex/gender bias has been showed in the outcome of many acute illnesses [6].

Major trauma is one of the leading causes of death and disability worldwide. It refers to severe injuries that have the potential to cause prolonged disability or death and require urgent specialised care. Major trauma is typically defined by an Injury Severity Score (ISS) ≥ 15. Sex/gender bias remains less studied and poorly understood in this context. While some authors have suggested a better prognosis in women due to a potentially protective role of female hormones [7, 8], emerging evidence indicates that differences in care delivery are also present. Recent studies have reported sex/gender inequity in both the management of coagulation disorders and in the triage of major trauma, where the severity of injuries in women may be under-recognised [9–11]. As a result, injured women appear less likely to be transported to, or admitted directly into, trauma centres, which may contribute to increased morbidity and mortality [12].

The aim of this scoping review is to examine the current evidence of sex/gender bias in major trauma care.

## Method

### Study setting and eligibility criteria

We performed a scoping review following the Preferred Reporting Items for Systematic Reviews and Meta-Analyses (PRISMA) guidelines (Liberati et al., 2009) [13], using the PRISMA-ScR Checklist (Extension for Scoping Reviews). The protocol was registered in August 2022 (Open Science Framework, https://osf.io/wvuj8, Registration 10.17605/OSF.IO/WVUJ8) [14]. This scoping review aims to explore sex and gender-based differences in the emergency management of severely injured patients, based on an extensive review of the literature.

All studies that included biological sex and gender in their analysis, within the context of major trauma, were evaluated from the first of January 1946 to the 31 of December 2024. We included original research studies, systematic reviews, letters, guidelines, but excluded conference abstracts and comments about studies to ensure availability of full data and methodological details. No geographical or language restrictions were applied. Studies involving children under 16 were excluded to focus on adult major trauma population.

### Information sources, search equation and selection process

We conducted a literature search in the following electronic database: Medline Ovid ALL, Embase, CINAHL with Full Text, Web of Science Core Collection, Cochrane Central Register of Controlled Trials, with search criteria including keywords and mesh terms: gender, sex, major trauma, wounds and injuries. The search strategy with syntax adapted for Medline was the following: (exp Multiple Trauma/OR (((multiple OR major) ADJ1 (trauma* OR injur* OR wound* OR fracture*)) OR polytrauma* OR "trauma care”).ab,ti,kf.) AND (exp Gender Identity/OR Gender Equity/OR Sex Characteristics/OR Sex Factors/OR Sexism/OR (((Gender OR Sex OR Sexes) ADJ4 (difference* OR bias OR characteristic* OR influence* OR disparit*)) OR "gender specific").ab,ti,kf.) NOT ((exp Child/OR exp Infant/OR exp Adolescent/) NOT exp Adult/). The search strategies adapted for each database are detailed in Supplementary File 1.

Reference lists of included articles were screened for additional publications. Three reviewers (CG-LM-AM) independently selected articles based on the inclusion criteria, with the final search conducted on December 28, 2024. The three reviewers classified the articles and extracted data with Rayyan software (QCRI software, https://www.rayyan.ai) [15]. Any disagreements were resolved by consensus or by decision of a fourth reviewer (FXA).

### Data collection and data analysis

We collected data using a semi-structured form that included study design, country of study, sex of the first and last authors, sex or gender disaggregated analysis performed, main topic, main objective, and main results of the study. We did not perform statistical analysis due to the study design. The data extraction was performed independently by two reviewers (LM-FXA). No critical appraisal of included sources of evidence was performed, as it was not required for this scoping review.

### Outcomes, intervention of interest and synthesis of results

Due to the study design of a scoping review, we did not identify a specific outcome to analyse.

We highlighted several topics of interest: mechanism of injury (MOI); trauma care, triage and transfer to a trauma centre; therapeutic interventions; traumatic haemorrhage management, trauma-related outcomes and traumatic brain injury (TBI) management. Data were grouped and analysed according to these topics of interest, while also considering the temporal sequence of events in trauma care, including patient triage and orientation, pre-hospital management, and in-hospital management. Patient age was also considered to compare outcomes across different age groups. Findings were also summarised descriptively in tables and figures to facilitate interpretation.

## Results

**Fig. 1:**
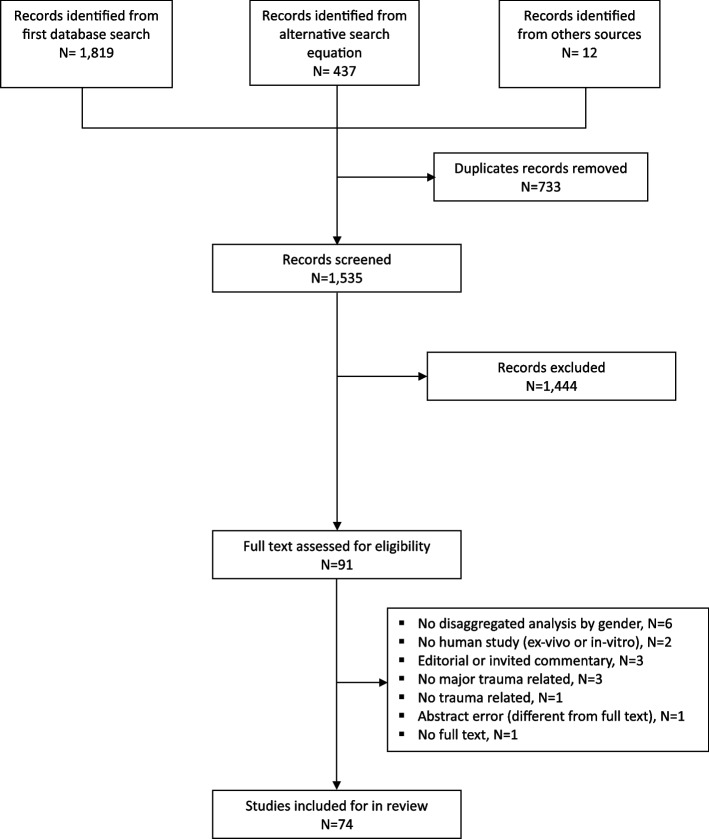
Study flow chart

This scoping review includes 74 articles. Figure 1 describes the study's flowchart. A total of 1,444 articles were excluded due to irrelevance to our topic or failure to meet inclusion criteria. After full-text review, an additional 17 articles were excluded for reasons such as lack of sex/gender disaggregated analysis, non-human studies, editorials, or studies not related to trauma.

**Fig. 2:**
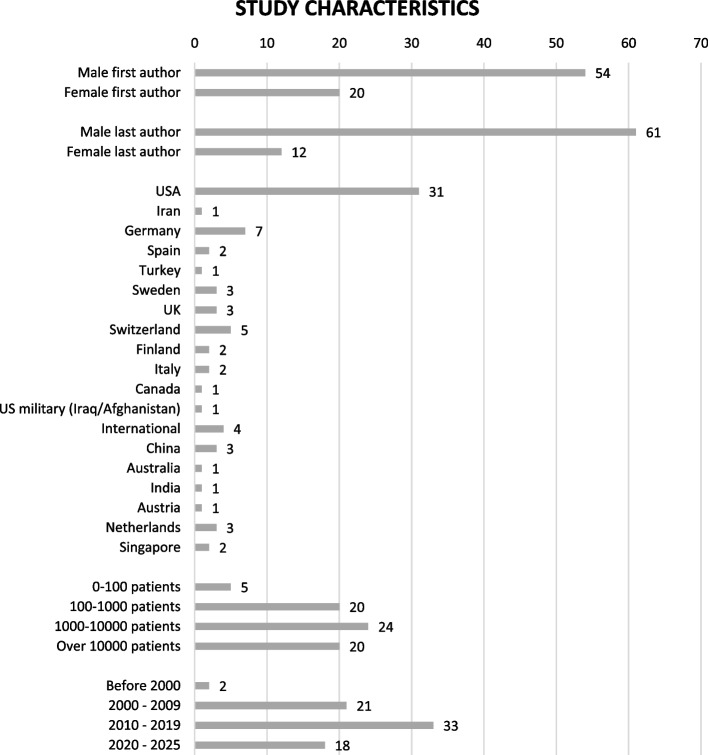
Study characteristics

Most of the studies were conducted in Europe and North America. All studies were observational and predominantly retrospective. One study reported secondary analysis of two randomised controlled trials with sex-disaggregated analysis that have not been reported previously [10]. A majority of the first and last authors were men, respectively 54 (73%) and 61 (82%). Most of articles published by female authors (as first or last author) are more recent, having been published after 2010. Figure 2 shows the study characteristics.

### Mechanism of injury and injury severity

Most studies reported that mechanisms of injury (MOI) differed between men and women (Table 1). Mitchell et al. [16] found that men were more likely to be victims of high-energy trauma (fall from height, motorcycle crashes) and were more likely to be victims of assault or stabbing injuries. In contrast, women were older and more likely to be victims of ground-level falls, pedestrian incidents, or passengers in a motor vehicle collision. They also were less severely injured than men, with lower Injury Severity Scores (ISS). For those not returning to independent living, women more frequently required nursing home admission. Hernández-Tejedor et al. [17] found that men were more likely to be victims of motorcycle crashes, work-related accidents and assaults. However, women admitted to the Intensive Care Unit (ICU) had higher ISS than men. Kahramansoy et al. [21] and Hernández et al. [17] found that women attempted suicide more frequently than men. Nutbeam et al. [24] found that women were more likely to be trapped in motor vehicle crashes than men (16% and 9%, *P* < 0.001). In this study, women were older than men (mean age 52 versus 44 years *P* < 0.001) and had less severe injuries (median ISS 17 versus 19, *P* < 0.001). However, women had more spinal cord and pelvic injuries, and men more head, thoracic and limb injuries. George et al. 2003 [18] showed that in the United States of America (USA), women were less likely to suffer from penetrating injuries than men. Motor vehicle crashes were the primary cause of injuries among men, while falls were the leading cause of injuries among women. Oström et al [20]. analysed alcohol consumption in traffic victims by sex/gender and found that men were more affected than women (32% versus 10% respectively). Ansorge et al. [27] and Balet et al. [28] showed that women were less likely to sustain high-energy trauma but had more unstable pelvic ring fractures (type B or C) than men after high energy trauma. Toimela et al. [29] also found a higher incidence of pelvic fractures in women. Two studies by Lassila et al [25]. and Marchesini et al [26]. found that women were more likely to suffer from spinal injuries.

**Table 1:** Main results by key topics

	**Higher prevalence in men**	**Higher prevalence in women**
Characteristics of injuries		
Mechanism of injury	- Assaults [16–19] - Penetrating injury [16, 18] - Motorcycle [16, 17]/motor vehicle accidents [18] - Vehicle accidents related to alcohol consumption [20] - Falls from higher heights [16]	- Self-inflicted injuries, suicide [9, 17, 21] - Blunt trauma [22, 23] - Passenger in a motor vehicle crash - Trapped in a motor vehicle accident [16, 24] - Falls from lower heights [12, 16, 18]
Severity of injury	No evidence of clinically significant difference in injury severity (ISS). Some studies showed a higher ISS in men [16, 24], others showed a higher ISS in women [17, 23], while others found similar scores [12, 22]	
Location of injury	Head [12, 24]/Chest [12, 24]/Abdomen [12]/Limbs [24]	Spinal cord [24–26]/Pelvis [24, 27–29]
Age	Younger [12, 16, 22–24]	Older [12, 16, 22–24, 30]
Coagulation disorders	- Greater hypercoagulability in thromboelastography in women, with no difference in standard laboratory assays [19, 23] - Lower fibrinogen levels at admission in women [31] - Greater hyperfibrinolysis in older women [32]	
Orientation and Triage		
Trauma centre admission*	Greater probability of transport [9, 12, 33, 34] and direct trauma centre admission [9, 35–37] in men	
ICU admission	Greater probability of ICU admission in men [38]	
Triage	Higher undertriage rates among women with moderate to severe injuries, except for penetrating trauma [12]	
Prehospital management		
Analgesia/Intubation/CPR/Vasopressors	No overall difference [17, 39], except in the study by Schauer et al. [40], where women received less analgesia	
Fluid resuscitation**	Larger volumes of fluid resuscitation in men [41, 42]	
Tranexamic acid	Nutbeam et al. [10] showed that men were more likely to receive tranexamic acid, despite similar effectiveness in men and women	
In-hospital management		
Emergency surgery	No clear evidence of differences. Trentzsch et al. [39] study reported more surgery in men. Hernández-Tejedor et al. [17] found similar management	
Mechanical ventilation	No difference [17]	
Fluid resuscitation**	Larger volumes of fluid resuscitation in men [41, 43]	
Blood transfusion	Men were more likely to receive blood transfusions [7, 39, 41]	
Outcomes		
Post-traumatic complications	Some studies reported higher rates of MODS and sepsis in men [2, 7, 13, 35, 44–46] Lee et al. [47] found similar complication rates between sexes while Stonko et al. [48] reported more complications in geriatric women	
Overall mortality	No evidence of a clinically significant difference in mortality. Some studies found higher mortality in men [8, 18, 45, 49–52], others in women [17], and several found similar mortality [16, 38, 42, 44, 47, 53–56].	
Mortality by age group	No consistent evidence of mortality differences by age and sex. Most studies found no difference [16, 38, 39, 42, 44, 47, 56–58]. Exceptions include: - Lower mortality in premenopausal women (Haider et al. [8]) - Higher mortality in men < 50 years (Wohltmann et al. [59]) - Lower mortality in women < 60 years (George et al. [49]) - Lower mortality in women < 45 years (Mostafa et al. [60]) - Higher mortality in older women (Dujardin et al. [32]) - Higher mortality in older men [61, 62]	
Cause of in-hospital death	Mostly MOF (61%) [63]	Mostly cerebral oedema (71%) [63]
Quality of life after polytrauma	Some studies reported poorer outcomes in women [64–67], whereas Gross et al. [68] found higher QoL in women	

*ISS* Injury Severity Score, *ICU* Intensive Care Unit, *CPR* Cardio-Pulmonary Resuscitation, *MODS* Multi-Organ Dysfunction Syndrome, *MOF* Multi-Organ Failure, *QoL* Quality of Life

^*^Only Rubenson et al. [9] and Gomez et al. [12] adjusted their results for injury severity

^**^ No adjustment was made for injury severity or BMI

Gomez et al. [12] found no difference in injury severity between men and women (median ISS 21 versus 20). However, men had a higher proportion of severe injuries to the head, chest, and abdomen than women. Women were older than men and more likely to be injured from ground level falls. Rubenson Wahlin et al. [9] found no sex/gender-based differences in injury severity and predominant anatomical regions of injury in Sweden. Women were older and more likely victims of self-inflicted injury, while men were more likely to be victims of assault. Gioffre-Florio et al. [30] focused on geriatric trauma patients and found that 62% were women.

### Coagulation disorders

Regarding coagulation disorders at admission, Schreiber et al. [19] reported that women were more likely to present early hypercoagulability on day one, based on thromboelastography and without any adjustment. However, standard clotting laboratory assays did not differ by sex. Coleman et al. [22] analysed the viscoelastic haemostatic profile of severely injured patients by sex/gender and also showed a more hypercoagulable state in women. They concluded this state conferred a survival benefit for women, despite finding a higher mortality rate in women. Pommerening et al. [23] also showed an hypercoagulability based on thromboelastography in premenopausal women, but without differences in standard laboratory assays and without differences in thrombotic events. Schoeneberg et al. [41] reported that deceased women had higher prothrombin ratios (PT ratios) than deceased men in matched analysis. Paydar et al. [31] showed that women had lower fibrinogen levels. Women were more likely to have hypofibrinogenemia than men in multivariate logistic regression. Dujardin et al. [32] found that older women were more likely to have hyperfibrinolysis and clotting factor consumption. Trentzsch et al. [39] found no sex/gender differences in coagulation parameters.

### Triage and transfer to a trauma centre (Table S2)

Holst et al. [35] found that half of the ED trauma related death were not admitted to a certified trauma centre. Women were less likely to be admitted in a trauma centre than men (adjusted OR: 0.83; 95%CI (0.70–0.99)). Pape et al. [38] found that men were more likely to be admitted in the ICU (OR: 1.21; 95%CI (1.05–1.39)). Gomez et al. [12] found that women were less likely to be admitted to a trauma centre by direct transport or by interfacility transfer; the OR adjusted for age, comorbidities, MOI and ISS was 0.87 (95% CI 0.79–0.96). Under-triage of women was relatively consistent across all age strata, except for the youngest and oldest strata (under 24 and over 85 years old). Under-triage of women was predominantly observed in intermediate or severe injury (ISS 15–24 and 24–47). Sex/gender disparity in trauma centre triage was observed for all injury mechanisms, except for penetrating injury.

Reilly et al. [36] compared trauma centre (TC) patients versus non trauma centre (NTC) patients and found that TC patients were disproportionately men (64.7% TC versus 47.2% NTC). Scheetz et al. [37] showed that 82% of men with an ISS >/= 16 were admitted to a trauma center, compared with only 60% of women with the same severity score, with women being on average older in the study population. They confirmed in a new study [33] in 2020 that advanced age, female gender, different ethnicity (especially Black ethnicity) and low average income are negative predictors of transfer to a trauma centre.

Rubenson Wahli et al. [9] found that men were more likely to be assigned prehospital top priorities and were more likely to be transported directly to a trauma centre than women. Concerning inter-facility transfer from rural communities, Quinn et al. [34] showed that elderly men were more likely to be transferred with shorter delays than elderly women.

### Prehospital and in-hospital emergency management

Trentzsch et al. [39] observed that women received similar prehospital interventions as men such as intubation, analgesia, use of catecholamines, fluid resuscitation, volume replacement and cardiopulmonary resuscitation. However, women received less surgical procedures than men during the acute care phase. Schauer et al. [40] studied the prehospital care of women in war zones (Iraq and Afghanistan) and found that local civilians women received less analgesia (we didn’t include the military population as it does not correspond to the population of our review). There was no difference in survival rates.

Regarding volume management, men were more likely to receive higher resuscitation volumes than women [41–43]. Schoenberg et al. [41] found that men received more prehospital and total fluid volume than women. Women were also less likely to receive blood transfusions. However, the authors only reported the number of packed red blood cells (PRBC) administered in the matched-pair analysis for men. Trentzsch et al. [39] showed that women were less likely to receive massive transfusion than men (6.9% versus 7.4%, *P* = 0.049). McKinley et al. [43] reported that women received less crystalloid volume and had a higher ISS. Regarding transfusion, women received fewer PRBCs in the first 12 h, but more PRBCs in total. Deitch et al. [7] found that pre and perimenopausal women received less blood products even though women presented with more major injuries and had higher ISS. Among patients admitted to ICU, Hernández-Tejedor et al. [17] found no statistically difference in emergency surgery, orotracheal intubation and length of mechanical ventilation between men and women. This study report only results from patients admitted to the ICU.

We found only two studies presenting sex-disaggregated analysis of randomised control trials assessing drug effectiveness in major trauma. Nutbeam et al. [10] presented a secondary analysis of the CRASH-2 and CRASH-3 trials [69, 70]. The effect of tranexamic acid (TXA) on traumatic death did not vary according to gender in the CRASH-2 and CRASH-3 trials separately (no heterogeneity, *P* value = 0.34). They also pooled the two RCTs and found the same treatment benefit in women than men with a reduction of early death of about 22%. Second, they analysed 216,316 injured patients in the UK trauma registry and found that women were less likely to receive prehospital TXA treatment than men when adjusted for the baseline risk of death from bleeding, OR: 0.35 (95% CI, 0.33–0.36). They also found that inequities were greater for older women and for women with low to intermediate risk of death from bleeding. Davenport et al. [71] reported prespecified sex-disaggregated analysis in the result of the randomised Cryostat-2 trial. They found no difference in the effectiveness of high-dose cryoprecipitate between men and women in severe traumatic haemorrhage.

### Prognosis and mortality (Table S3)

A few studies reported an increased risk of death for men compared to women after major trauma. Most of these studies, conducted by two research teams in North America using data from the National Trauma Data Bank, aimed to assess a potential protective effect of female hormones by stratifying analyses by age groups according to their presumed hormonal status. George et al. 2003 [49] observed that men had a higher risk of death than women in all age categories except for injured patients with blunt trauma after age 60. Haider et al. [8] showed that premenopausal women suffering from severe traumatic injury and shock were less likely to die than men; OR = 0.86; 95% CI (0.76–0.93). However, the result for postmenopausal women showed a non-statistically significant reduction in mortality, OR = 0.90; 95% CI (0.76–1.05), probably due to underpowered subgroup analysis. Wohltmann et al. [59] found higher mortality in men only in the < 50 age group (unadjusted OR 1.27, 95% CI (1.09–1.49)). Pecheva et al. [61] assessed frailty among older injured patients and found that men was a predictor of mortality with an adjusted OR = 1.6, 95% CI(1.09–2.49). One non-systematic review by Marcolini et al. [72] reported that male sex was associated with increased risk of in-hospital mortality using only a selection of studies. A meta-analysis by Liu et al. [50] also reported a slight increase in the risk of death for men, RR = 1.16, 95% CI (1.03–1.31). However, they did not consider the adjusted RR that was reported in the different studies and included only crude RR inducing potential confounding bias. Morris et al. [51] reported in 1990 that male sex/gender is a risk factor for mortality. Sammy et al. [62], in a systematic review and meta-analysis conducted in the UK, found 4 studies out of the 15 included that examined the impact of sex/gender on elderly trauma patients, with mortality seemingly higher in men. Yang et al. [52] showed a lower risk of in-hospital mortality for women among Chinese injured patients admitted to intensive care.

All other studies reporting in-hospital mortality found no sex-related difference in mortality, irrespective of the study region (North America, Europe, Asia) (Table S3) [16, 38, 42, 44, 47, 53–56]. Furthermore, female sex/gender was not significantly associated with mortality in the different age strata in most studies [16, 38, 39, 42, 44, 47, 56, 57].

Nutbeam et al. 2022 [24] were specifically interested in vehicle accidents. They found that women had a statistically significantly lower survival rate, although the difference was small (94.0% versus 94.6%, *P* = 0.001).

Several studies [2, 7, 13, 35, 44–46] showed that men had a higher risk of multi-organ dysfunction syndrome (MODS) and sepsis than women. Oberholzer et al. [63] found that most women died from cerebral oedema (71%) and most men died from multi-organ failure (MOF) (61%). They concluded that women were at lower risk of sepsis and MOF than men without any adjustment. In propensity score matching analysis, Lee et al. [47] found no differences in posttraumatic complications and mortality. Stonko et al. [48] identified female gender as a risk factor for posttraumatic complications in a geriatric population. Weuster et al. [73] found that female gender was a risk factor for developing accidental hypothermia.

A few studies have examined the link between sex/gender and quality of life after major trauma. Gross et al. [68] found a higher quality of life for women after 1 and 2 years of follow-up. On the other hand, Llaquet Bayo et al. [64] showed that woman was associated with poorer quality of life, Tan et al. [65] found worse functional outcomes for women and Ringburg et al. [66] reported female sex/gender as a predictor for long-term disability. Recently, a systematic review by Lotfalla et al. [67] reported that female sex was a predictor for worse health related quality of life.

### Traumatic brain injury

We found 6 studies focusing on exclusively traumatic brain injury (TBI) (Table S4). Albrecht et al. [74] found no sex-based differences in mortality among older adults with isolated TBI. Yeung et al. [75] found the same result in younger women aged 12 to 45 years. Davis et al. [76] found no sex-based differences in survival for premenopausal women. Ng et al. [77] and Kraus et al. [78] found a poorer outcomes in women. Kraus et al. [78] observed that women were 1.6 times more likely to have poor outcomes than men with mortality at 18 months adjusted for age, Glasgow Coma Score (GCS) on admission, penetrating versus blunt injuries and the presence of multiple traumas. In contrast, Mair et al. [79] found a lower overall mortality in women after TBI (which was statistically significant only in postmenopausal women).

## Discussion

This scoping review suggests that injured women are more likely to be older, victims of low-energy trauma such as ground level falls and self-inflicted injury. Men are more likely to be victims of high-energy trauma such as motor crash vehicle and assault. In terms of trauma care, women are less likely to be transported to a trauma centre, less likely to receive TXA and blood transfusion. We found no evidence of a relevant difference in mortality between women and men.

Under-triage of injured women appears to be a consistent theme across included studies and occurs despite comparable or greater injury severity in some populations. Interestingly, most studies included in this review used databases from level 1 trauma centres. In this specific context, the under-triage rate is higher for women, thus creating survival bias or selection bias. Selection bias could explain the lower mortality in women observed in some studies because women who died in non-trauma centres were not included in the analysis, or because severely injured women were less likely to be transferred to level 1 trauma centres. As the studies included in our review did not provide information on the management of patients not transported to a trauma centre, it is difficult to assess whether these patients received adequate trauma care in other centres.

The differences in mechanism of injury could explain some of the differences in trauma care and under-triage. Most emergency clinicians and paramedics in dispatch centres use mechanism of injury to subjectively identify major trauma, which deprioritises the types of major trauma more commonly sustained by women, such as low-level falls and pedestrian injuries. Differences in clinical presentation between men and women, particularly among older trauma patients, may bias the initial assessment performed in the field. The interaction between dispatch algorithms, responder perception, and potentially physiological differences may contribute to the lower rates of trauma team activation and trauma centre transfer for women. These findings highlight a need for triage tools that are validated across sex and gender and that do not rely excessively on male-normative injury patterns.

Regarding the other results, there appears to be no significant difference in coagulation disorders, except for a possible tendency toward hyperfibrinolysis in women. Women receive fewer blood transfusions, less fluid volume and less prehospital tranexamic acid. Adjusted mortality appears similar between women and men, with conflicting evidence of increased mortality in men in some studies. Organ failure and sepsis appear less frequent in women.

These disparities should not be interpreted as inevitable consequences of biological difference. The hypothesis that they are caused by preventable aspects of trauma care systems, such as triage algorithms, dispatch protocols, clinician behaviour and gender bias, should be seriously examined. These factors influence the prognostic differences observed between men and women. Observed disparities in access to trauma care, fluid resuscitation, and use of tranexamic acid suggest that structural and operational factors, rather than innate sex-based physiology, may underpin much of the inequity reported.

All studies included in this review had a high risk of bias, including selection bias, confounding, generalisation bias, statistical overfitting, collider bias and misclassification by sex, except the sex-disaggregated analysis of randomised CRASH-2 & 3 and CRYOSTAT trials. Observational studies may be biased by confounding factors. This review shows differences between men and women in MOI and to a lesser extent in injury severity. Hence, crude analysis is not appropriate to assess sex-based differences in mortality. The selection of confounding variables used for adjustment is a critical consideration. Many studies reported in this review included too many covariates that might lead to overfitting and bias toward the null the association between the predictor and the outcome. Most studies did not provide details and justification for multivariate models and covariate selection. Studies by George et al. and Haider et al. showed an increased risk of death for men in North America but included complications and mechanism of injury as covariates. This may lead to collider bias and overadjustment, as the covariates are in the causal pathway between the exposure and the outcome.

Some studies presented in this review attempted to evaluate a potentially protective effect of female hormones. This review found no evidence supporting this hypothesis. Most studies presenting differences in mortality by age categories (pre or postmenopausal age) performed subgroup analysis without heterogeneity testing. These subgroup analyses led to underpowered analyses with inconclusive results and false conclusions as in the study by Haider et al. [8] Furthermore, the division into different subgroups (pre- or postmenopausal) was based on age rather than the hormonal status of the included women leading to misclassification.

This review has some strengths and limitations. We conducted a rigorous literature search with a clear research strategy. However, this review may have failed to identify all studies of interest. The high number of publications about posttraumatic stress disorders led us to restrict the research equation to multiple or major trauma. We prespecified the main topics to search in the protocol. We may have missed some information not prespecified in the full text analysis. We restricted our analysis to sex/gender disparity in mortality and in mechanisms of injury.

Future studies assessing sex/gender bias in major trauma must be conducted prospectively. Selection of covariates to be included in a multivariate models should be rigorous, using dedicated methods, such as penalised regression or Least Absolute Shrinkage and Selection Operator described by Tibshirani et al. [80] Future research should prioritise the development and validation of sex- and gender-sensitive triage tools and major trauma protocols. Trauma systems should consider audits of access and treatment by sex and gender, particularly focusing on time-critical interventions such as tranexamic acid administration, trauma team activation, and access to specialist centres. Implementation science strategy and working on unconscious bias with healthcare providers may be necessary to ensure that changes in policy translate into measurable improvements in care equity. It is also necessary for future research to consider the current diversification of gender, which is no longer binary (e.g., transgender, non-binary, genderfluid individuals, etc.), as well as the evolving roles in society, to ensure that everyone can be represented. The findings of this review reinforce the need to embed sex and gender analysis across all stages of trauma system design and research to promote fairer and more effective care delivery.

## Conclusion

Women are less likely to receive trauma care and be transported to a trauma centre. While differences in injury mechanisms may play a role, these disparities appear to reflect modifiable features of trauma systems, including prehospital triage processes, transfer pathways, and clinical decision-making. Addressing them likely requires system-level changes, including sex- and gender-sensitive approaches, to ensure equitable access to specialist trauma care.

## Supplementary Information

Supplementary Material 1. [81–85].

## Data Availability

All data generated or analysed during this study are included in this published article, its references and its supplementary information files.
