# Supplementary material for: Sex and gender bias in major trauma care: a scoping review
Source: Scand J Trauma Resusc Emerg Med. 2026 Mar 24;34:84. doi: 10.1186/s13049-026-01596-3 (PMC13134334; doi:10.1186/s13049-026-01596-3)
Supplement: Supplementary file 1 — Supplementary Material 1. [81–85]. [file 13049_2026_1596_MOESM1_ESM.docx]

**Supplementary material**

Supplement 1. research equations

We conducted a literature search in the following database: Medline Ovid ALL, Embase, CINAHL with Full Text, Web of Science Core Collection, Cochrane Central Register of Controlled Trials.

The search strategy with syntax adapted for Medline was the following: (exp Multiple Trauma/ OR (((multiple OR major) ADJ1 (trauma* OR injur* OR wound* OR fracture*)) OR polytrauma* OR "trauma care”).ab,ti,kf.) AND (exp Gender Identity/ OR Gender Equity/ OR Sex Characteristics/ OR Sex Factors/ OR Sexism/ OR (((Gender OR Sex OR Sexes) ADJ4 (difference* OR bias OR characteristic* OR influence* OR disparit*)) OR "gender specific").ab,ti,kf.) NOT ((exp Child/ OR exp Infant/ OR exp Adolescent/) NOT exp Adult/).

The strategy with syntax adapted for Ovid: (exp Multiple Trauma/ OR (((multiple OR major) ADJ1 (trauma* OR injur* OR wound* OR fracture*)) OR polytrauma* OR "trauma care”).ab,ti,kf.) AND (exp Gender Identity/ OR Gender Equity/ OR Sex Characteristics/ OR Sex Factors/ OR Sexism/ OR (((Gender OR Sex OR Sexes) ADJ4 (difference* OR bias OR characteristic* OR influence* OR disparit*)) OR "gender specific").ab,ti,kf.) NOT ((exp Child/ OR exp Infant/ OR exp Adolescent/) NOT exp Adult/)

The strategy with syntax adapted for Embase: ('multiple trauma'/de OR 'multiple fracture'/de OR (((multiple OR major) NEXT/1 (trauma*OR injur* OR wound* OR fracture*)) OR polytrauma* OR "trauma care"):ab,ti,kw) AND ('gender and sex'/de OR 'gender'/exp OR 'gender bias'/de OR 'sex difference'/exp OR ((Gender OR Sex OR Sexes) NEAR/4 (difference* OR bias OR characteristic* OR influence* OR disparit*)):ab,ti,kw) NOT ('juvenile'/exp NOT 'adult'/exp) NOT ('conference abstract'/it OR 'conference review'/it)

We make the following observations for considering the previous search equation. Trauma* alone is excluded because it brings a lot of references on PTSD or psychological trauma. We preferred to add a proximity search like “multiple” close to trauma*, injur*, wound*, fracture*. Fracture multiple is a specific term of "Multiple Trauma" [Mesh], but taken separately on Embase because it is not the same hierarchy. “Sexism/” is the descriptor used for “gender bias”. “Trauma center” not retained, because it adds too many irrelevant articles.

sTable 1. Characteristics of the study about mechanism of injury, injury pattern and coagulation disorders by gender.

| Author | Study design | N | Country | Sex of authors  First / Last | | | Main objective of the study | Outcome / Main result |
| --- | --- | --- | --- | --- | --- | --- | --- | --- |
| Schreiber et al, 2005^28^ | Prospective cohort | 65 | USA | M | M | Assess the coagulation changes and determine whether there is a difference between men and women (crude analysis) | | Thromboelastography (TEG) /  mean rTEG was 1 minute earlier in women than in men. Standard clotting parameters did not differ by gender. |
| Paydar et al. 2018^32^ | Retrospective observational | 855 | Iran | M | M | Examined the feasibility of the use of different clinical and para-clinical variables in predicting the need for fibrinogen replacement therapy. | | Women have low level of fibrinogen. Multivariate logistic regression showed 60% less hypofibrinogenemia (<1.0) in men compared to women. |
| Hefele et al. 2019^81^ | Prospective cohort | 20 | Germany | F | M | phenotype the human immune response following injury and to identify risk factors of trauma induced injury. | | Increase of IL-17a on Th17 cells and CD4+ tregs (which increase the risk of sepsis). Male have higher ratio Th17/CD4+ and Treg/CD4 (higher risk of sepsis). However MCF extem and fibtem higher in male. |
| Sperry et al. 2008^57^ | Prospective observational | 80 | USA | M | M | similarly injured males and females would differ in their early cytokine expression profile and that these differences would be associated with distinct clinical outcomes after injury. | | IL-6 serum levels were statistically higher in males relative to females (*p* = 0.008). Gender Male is associated with MOF (but not to Nosocomial infection). Of note= less intervention and blood in women… |
| Dossett et al. 2008^82^ | Prospective observational | 991 | USA | F | M | Assess if endogenous estrogens would be associated with mortality in critically inured patients | | Deaths increased by 3 times with estradiol. No difference between gender. estrogens may simply reflect global illness |
| Coleman et al. 2019^29^ | Prospective observational | 464 | USA | F | M | Examine viscoelastic hemostatic profile of severely injured patients by sex | | Female were older, arrive later in-hospital, more blunt trauma, higher ISS, lower SBP, higher BE, lower Hb. Authors conclude to hypercoagulable state in women with higher MA and angle in TEG. LY30% was similar but % of LY30>3% was higher in male (26% Vs 15%). Despite all baseline characteristic women did not receive more blood, neither massive transfusion suspecting under-care. Mortality was higher in women (33% versus 25%). Surprisingly, authors concluded no difference in this underpowered study. And concluded to ”female sex conferred a survival benefit in the setting of decreased MA” |
| Hernández-Tejeda et al, 2008^17^ | Prospective cohort | 953 | Spain | M | M | Difference by gender in injury mechanism and injury pattern in patients admitted in ICU (standardization by TRISS) | | Maximum AIS and ISS / MOI  Difference in MOI by gender. Women were more severe, presented more severe TBI. Higher mortality in women without difference in TRISS. |
| Kahramansoy et al, 2013^18^ | Retrospective cohort | 6,185 | Turkey | M | M | MOI in rural areas with a gender-based evaluation (univariate analysis) | | MOI, Injury body regions, ED outcome / Only 13% all of trauma were women, 19% of motor crash accident, women were exposed to more severe trauma; high rate of suicide attempt. |
| Schoeneberg et al, 2015^31^ | Retrospective cohort | 1,073 | Germany | M | M | Assess gender-specific differences in the treatment and in laboratory tests (subgroups analysis by survived and deceased men and women with matching by AIS, age, and ISS) | | MOI, Coagulation assays /  Women were older with different MOI,  Value of coagulation assay were higher in women. |
| Ostrom et al. 1995^21^ | Retrospective observational | 597 | Sweden | M | M | Analyze gender differences among traffic fatalities and alcohol consumption (Autopsy). | | Blood alcohol detected in 10% of deceased women (32% in men). No increase by 10y. Drunken driving and alcohol abuse in traffic is still mainly a male problem. |
| Nutbeam et al, 2022^19^ | Retrospective cohort | 70,027 | UK | M | M | Assess the probability of entrapment, the frequency of injury and outcome by gender (adjusted on ISS, age, comorbidities, GCS) | | Entrapment, Injury regions/  Women were more frequently trapped; Head, Thoracic and limb injuries for men; Pelvic and spine injury for women; No difference in mortality |
| Ansorge et al. 2023^22^ | Retrospective observational | 434 | Switzerland | M | M | Determine the gender-specific and global incidences of high-energy type B or C pelvic ring injury | | Women less likely to sustain high energy trauma; But women presented more instable pelvic ring fracture B or C than men (18% vs 8%) during high-energy trauma. |
| Balet et al. 2023^23^ | Retrospective observational | 2,790 | Switzerland | M | M | Describe the use of pelvic binder in the pre-hospital setting and identify pre-hospital factors associated with unstable pelvic fractures. | | No difference in use of pelvic binder by sex. Women more likely to have unstable pelvic fracture Type B or C. |
| Toimela et al. 2021^24^ | Retrospective observational | 1,593 | Finland | M | M | Analysed the changes in injury mechanisms that lead to high-energy pelvic trauma | | Pelvic ring fracture patients were more likely to be female (39% vs 22%, *p* < 0.001), to be more severely injured (NISS 35.2 vs 30.4, *p* < 0.001), injured due to a high fall (41% vs 25%, *p* < 0.001), to have self-inflicted injuries (23% vs 8%, *p* < 0.001), and to have higher 30-day in-hospital mortality (13% vs 9%, *p* = 0.005). |
| Gioffre-Florio et al. 2018^27^ | unknown | 4,554 | Italy | F | M | Assess prevalence of geriatric trauma, mortality and sex difference | | Elderly (>65) represents 20% of overall trauma. 60% have ISS>15. 62% were women. Head injury is predominant. (mortality in ED 0.06%, no data of in-hospital mortality) |
| Lassila et al. 2024^25^ | Retrospective observational | 2,529 | Finland | M | M | Determine the incidence and severity of spine injury among severely injured patients (ISS/NISS > 15) and compare the demographics between patients with or without spine injury | | Female gender is overrepresented in TSI (traumatic spine injury) patients with a self-inflicted mechanism. |
| Marchesini et al. 2024^26^ | Retrospective observational | 1,545 | Italy | M | M | Define demographics, patterns of injury and clinical data in patients with traumatic brain injury and concomitant upper cervical injury | | Women are more likely to present a concomitant cranial and upper cervical spine injury. |
| Pommerening et al.2014^30^ | Prospective observational | 795 | USA | M | M | Determine if rapid [thrombelastography](https://www.sciencedirect.com/topics/biochemistry-genetics-and-molecular-biology/thromboelastography) (rTEG) coagulation profiles differ by gender | | Females were older with higher ISS (ISS 22 vs 20; *P* = .03) and were more frequently injured by blunt mechanisms (88% vs 69%; *P* < .001). Premenopausal women showed a hypercoagulability based on thromboelastography at the time of admission and for the initial 12 hours after injury, but without difference in standard laboratory assay and without difference in thrombotic events |
| Dujardin et al. 2024^33^ | Secondary analysis of a prospective multicentre trial | 1,345 | International (EU) | M | F | Compare markers of coagulation between male and female trauma patients across different ages, to see if female sex may provide a survival benefit | | Older women were more likely to have hyperfibrinolysis and clotting factor consumption.  Females after their reproductive age have a higher mortality after trauma compared with younger females and males |

TEG: Thromboelastograph; ICU: Intensive care unit; TRISS: Trauma Score and Injury Severity Score; AIS: Abbreviated Injury Scale; ISS: Injury Severity Score; MOI: Mechanism of injury;

sTable 2. Characteristics of the study about trauma care, trauma centre admission and treatment by gender.

| Author | Study design | N | Country | Sex of authors  First / Last | | Main objective of the study | Outcome / Main result |
| --- | --- | --- | --- | --- | --- | --- | --- |
| Deitch et al, 2007^7^ | Prospective cohort | 5,192 | USA | M | M | Relationship between gender and early response to injury (Adjusted for age only) | Blood lactate at 30 min and need for early transfusion /  Women received less blood RBC unit with higher ISS; Lactate level was lower in women; Mortality was higher in women (NS) |
| Gomez et al. 2012^12^ | Retrospective cohort |  | Canada | M | M | Relationship between gender and access to TC (adjusted for age, comorbidities, MOI, ISS) | TC admission /  Women are less likely to be admitted in a TC, OR: 0.87; 95% CI (0.79-0.96) |
| Quinn et al. 2022^40^ | Retrospective observational | 1,535 | USA | M | M | Evaluates the relationship between inter-facility transfer time and outcomes in elderly trauma patients | Shorter dwell time was associated with men, higher ISS and higher mortality. No stratification on gender was performed for mortality. |
| Reilly et al. 2004^37^ | Retrospective observational | 103,725 | USA | M | M | Compare injured patients' outcomes in New York City's level 1 trauma centers and nontrauma centers. | Patients in trauma center were younger than those in non-TC and disproportionately men (65% versus 47%) |
| Schauer et al. 2019^41^ | Retrospective observational | 20,018 | US military (Iraqi/ Afghanistan) | M | M | Compare injury patterns and interventions performed in the prehospital, combat setting among females versus males. | No difference in mortality. Women (host civilian) were less likely to receive morphine and ketamine (probably military too, but underpowered study) |
| Scheetz et al. 2004^38^ | Retrospective observational | 5,712 | USA | F |  | admission of adults with differing levels of injury severity were compared, based on patient age and gender. | with ISS >or= 16, younger men were most likely to be admitted to a TC (82%), and older women were least likely to be admitted to a TC (60%). |
| Scheetz et al. 2020^39^ | Retrospective observational | 33,696 | USA | F | M | examine sociodemographic predictors of trauma center (TC) transport of severely injured older adults. | Predictors of non-TC transport were as follows: older age groups (OR 0.92, CI 0.76-1.11; OR 0.79, CI 0.64-0.96; OR 0.77, CI 0.63-0.95), females (OR 0.65, CI 0.57-0.74), Black and “other” race (OR 0.75, CI 0.0.56-1.0; OR 0.96, CI 0.77-1.20), lower median household income (OR 0.76, CI 0.62-0.93; OR 0.86, CI 0.71-1.05) |
| Rubenson Wahlin et al. 2016^9^ | Retrospective cohort | 383 | Sweden | F | F | Gender-related differences in prehospital trauma care | Prehospital priority, Transport to TC /  Males were more likely to receive highest level of priority. Women were less likely to be transport in a TC. |
| Holst et al, 2016^35^ | Retrospective cohort | 3,971 | USA | F | M | Undertriage in trauma-related death in urban and rural setting (multivariate model) | TC versus non-TC /  Women were more undertriaged, OR: 0.83, 95% CI [0.70–0.99]) |
| Ingram et al, 2022^11^ | Retrospective cohort | 28,332 | USA | F | F | sex-based differences in timeliness of trauma care (PSM: age, ISS, mechanism, and injury type with caliper width 0.5) | Discharge disposition including mortality / Longer ED LOS and delays in femur or pelvic repair for women; longer delays in trauma care and more long-term facility for women. |
| Yücel et al. 2006^83^ | Retrospective observational | 4,527 | Germany | M | M | Prognostic model to predict massive transfusion. | Men were more likely to receive massive transfusion in multivariate model: OR=1.5 |
| McKinley et al. 2002^43^ | Retrospective observational | 58 | USA | M | M | Should women respond better to standardized resuscitation compared with similarly severely injured men | Women presented higher ISS, BD; received less PRBC in the 12h (but more PRBC in total), and less volume crystalloid (for the same goal of oxygen delivery index >600ml.min) |
| Nutbeam et al, 2022^10^ | Secondary analysis of RCT with sex-disaggregated analysis / cohort | 32,948 /  216,364 | International / UK | M | M | TXA effectiveness and administration by gender (adjusted for baseline risk of death from bleeding, MOI, age) | Early death and Prehospital TXA /  No heterogeneity in TXA effectiveness; Women were less likely to receive prehospital TXA |
| Davenport et al. 2023^46^ | interventional, randomised, open-label, parallel-group controlled, multicenter study | 1,531 | International (UK/USA) | M | M | Assess whether survival could be improved by administering a high dose of cryoprecipitate to  patients with trauma and bleeding with activation of a major hemorrhage protocol | No difference in the effectiveness of high-dose of cryoprecipitate between men and women in severe traumatic haemorrhage (prespecified sex-disaggregated analysis). |

RBC: Red Blood cells; ISS: Injury Severity Score; TC: trauma centre; PSM: Propensity score Matching; ED: Emergency department, LOS: Length of stay; RCT: Randomised control trial; TXA: Tranexamic acid; MOI: Mechanism of Injury

sTable 3. Characteristics of the study about mortality and complications by gender.

| Author | Study design | N | Country | Sex of authors  First / Last | | Main objective of the study | Outcome / Main result |
| --- | --- | --- | --- | --- | --- | --- | --- |
| Morris et al. 1990^52^ | Retrospective observational | 199,737 | USA | M | F | influence of the following host factors: age, gender, and preinjury medical conditions, on mortality stratified by injury severity. | Stratified analysis (unadjusted) Men Male younger, women older; higher mortality for men |
| Oberholzer et al, 2000^64^ | Retrospective observational | 1,276 | Switzerland | M | M | Association between sex and posttraumatic complications and mortality in severely injured patients (adjusted for age, ISS) | Sepsis, MODS (PCT, IL-6, IL-10) /  No difference with ISS<25; Higher Sepsis and MODS in men with ISS>25 |
| Gannon et al. 2002^55^ | Retrospective observational | 22,332 | USA | M | M | Association gender and mortality (adjusted race, RR, SBP, RTS, injury type, comorbidities) | In-hospital Death /  No difference in mortality by gender |
| George et al. 2003^47^ | Retrospective observational | 7,438 | USA | M | M | Association gender and mortality (adjusted for ISS, age, race, MOI, comorbidities, complications, and stratification by age) | In-hospital Death /  Increase mortality for men < 50 y |
| Newman et al. 2022^56^ | Retrospective observational | 1,336 | USA | F | F | Examines geriatric trauma patients who are deemed unexpected survivors (TRISS <.5) to identify factors that may confer a survival benefit. | No influence of gender |
| Pecheva et al. 2020^49^ | Retrospective observational | 819 | UK | F | M | Assess the impact of frailty on outcomes in older patients who experience major trauma. | Mortality increased with increased frailty. Adjusted OR of death 1.65 for men compared to women. |
| Pena et al. 2017^84^ | Prospective observational | 39 | USA | M | M | tested whether X-linked cellular mosaicism, which is unique to females, could represent a genetically based mechanism contributing to sex-related immuno-modulation following trauma. | Skewing X-Chr inactivation occurred with increased injury severity and with time. Increased X-chr skewing associated with infection. |
| George et al. 2003^20^ | Retrospective observational | 175,702 | USA | M | M | Association gender and mortality (adjusted for ISS, age, race, comorbidities, complications) | In-hospital Death /  Increase mortality for men ≥ 50 y |
| Sperry et al. 2008^57^ | Retrospective observational | 1,036 | USA | M | M | Assess protective effect afforded by female after traumatic injury (adjusted for: ISS, age, Transfusion, BMI, GCS, hypotension, base deficit, head AIS and stratification by age categories). | MOF, Infection, In-hospital Death /  No difference in mortality by gender  No difference by age categories (≥50 y) |
| Wohltmann et 2001^48^ | Retrospective observational | 20,261 | USA | M | M | The purpose of this study was to evaluate whether severely injured women have a survival advantage over men. Evaluation of the effects of gender dimorphism on survival in trauma patients. | No difference between men and women. “Significant difference in age <50: **Unadjusted** OR of death for men:1.27 (1.09-1.49). |
| Yang et al. 2014^54^ | Retrospective observational | 858 | China | M | F | Our research attempted to determine whether the female sex is associated with a survival advantage among severely injured Chinese trauma patients admitted in ICU. | Among all trauma patients, females had a significantly lower risk of in-hospital mortality compared with males (OR, 0.41; 95% confidence interval [95% CI], 0.20 – 0.85). Kaplan-Meier showed that difference in mortality appeared later (>10 days). |
| Samuelsson et al. 2015^58^ | Retrospective observational | 4,320 | Sweden | F | M | evaluate the association between sex and 30-day mortality in a Swedish general ICU cohort in which the study population was dichotomized at the median age for female menopause (45 years) to see if a premenopausal female hormone profile is associated with better outcome following intensive care. | No association between sex and mortality in trauma regardless of premenopausal age or not. Globally, no association between sex and mortality in Swedish ICU (N=127,254) |
| Haider et al. 2009^62^ | Retrospective observational | 681,730 | USA | M | M | Sex-based differences in developing complications and in risk of mortality (adjusted on age, race, ISS, AIS head, SBP, MOI, penetrating injury, insurance status) | In-hospital death, ARDS, Infection, PE, DVT, UTI, ARF/  Increase mortality and complications in men. Increase risk of death for women with complications. |
| Haider et al. 2010^8^ | Retrospective observational | 48,394 | USA | M | M | Compare mortality outcomes between preadolescent children, hormonally active adults, and elderly patients who are largely postmenopausal. (Stratification by age categories and adjusted for ISS, RTS, AIS head, MOI, race, insurance status). | In-hospital death /  Crude analysis showed increase of death in all age categories. Adjusted analysis showed an increase of death in “hormonal female” only. |
| Mitchell et al, 2012^16^ | Retrospective observational | 6,763 | Australia | F | M | Assess epidemiologic profile and trauma outcome in men and women (univariate analysis) | ISS, AIS, LOS /  Difference in MOI, longer LOS for men, higher ISS for men; no difference in mortality. |
| Trentzsch et al, 2014^42^ | Retrospective observational | 10,334 | Germany | M | M | Association between MOF and sepsis in females of reproductive age (age subgroups analysis with univariate analysis) | MOF, Sepsis /  Increase of risk of MOF and sepsis in men; Non significant higher risk of death in women. No difference in mortality |
| Trentzsch et al, 2015^34^ | Retrospective observational | 7,774 | Germany | M | M | role of sex on rates of sepsis, MOF, and mortality (Matching on Age, Type of injury, AIS, SBP, stratification by age) | sepsis, MOF, in-hospital mortality/ Increase risk of MOF, sepsis, death for men independently of age categories. |
| Liu et al. 2015^51^ | Meta-analysis | 17 studiesstudies | China | M | M | Association gender and mortality | In-hospital death /  Increase mortality for men |
| Stonko et al. 2021^65^ | Retrospective observational | 614,496 | USA | M | M | Assess if overall mortality and the failure to rescue rate (defined as mortality after complication) worsen as age and injury severity increase. | Those with complications were more likely to be older, female, non-white, have a non-blunt mechanism (burn, penetrating, and other), a higher ISS, and worse vital signs on arrival |
| Wafaisade et al. 2011^63^ | Retrospective observational | 29,829 | Germany | M | M | to evaluate independent risk factors for posttraumatic sepsis | redictors for sepsis after severe trauma derived from multivariate analysis were male gender, age, preexisting medical condition, GCS score of ≤8 at the scene, ISS, AIS_THORAX_ score of ≥3, number of injuries, number of red blood cell units transfused, number of operative procedures, and laparotomy. |
| Sammy  et al. 2016^53^ | Systematic review and metanalysis | 15 studies | UK | M | F | Do differences in demographic, clinical and injury characteristics alter risk-adjusted mortality in older patients, 65 years and above, who have presented to the [Emergency Department](https://www.sciencedirect.com/topics/medicine-and-dentistry/emergency-department) or the Emergency Services with major trauma? | 4 studies (on 15) investigated gender as risk factor in geriatric trauma patients. Mortality seems increased for older men. |
| Verma et al. 2017^61^ | Retrospective observational | 781 | India | M | M | Assess predictors of mortality by gender in haemorrhagic shock (adjusted for age, sex, SBP, PTX, FAST, MOI) | In-hospital death  Increase survival in women |
| Marcolini et al, 2018^50^ | Non-systematic review | - | USA | F | F | Review about gender disparities in trauma | - |
| Weuster et al. 2016^66^ | Retrospective observational | 15,230 | Germany | M | M | to evaluate the current epidemiology of AH in major trauma patients. | we figured out that female gender is even a significant risk factor to become hypothermic at 33.0°C. it is yet not fully understood why female patients seem to be more susceptible to develop AH. The higher body-fat percentage in females and a higher body mass index protected against severe hypothermia.^27^ Charkoudian and Stachenfeld^33^ discussed endogenous reasons in women. Estrogens generally promote vasodilation, heat dissipation, and lower body temperature. |
| Joestl et al, 2019^59^ | Retrospective observational | 646 | Austria | M | F | Association between gender and outcome in multiple trauma patients (adjusted for age, ISS, hemodynamic instability) | In-hospital mortality /  No differences in mortality, MOF, ARDS. Higher risk of longer LOS and rehabilitation for older women |
| Mica et al. 2014^85^ | Retrospective observational | 651 | Switzerland | M | M | Test the impact of body mass index (BMI) and gender on infectious complications after polytrauma | Unadjusted analysis: Higher BMI showed lower mortality. Female showed lower mortality (unadjusted). |
| Pape et al, 2019^36^ | Retrospective observational | 6,865 | Netherlands | F | M | Association between gender and mortality (adjusted for age, ISS, SBP, intubation, MOI, AIS, and stratification by age) | In-hospital mortality /  No difference in mortality. More admission in ICU for men with lower ISS than women. |
| Lee et al, 2020^60^ | Retrospective observational | 10,012 | China | M | F | Association gender and mortality in major trauma (PSM on age, comorbidities, and injury regions) | In-hospital death /  No difference in mortality |
| Gross et al. 2019^67^ | Prospective cohort | 335 | Switzerland | M | M | Assess progress in gender-dependent longer-term outcomes after major trauma (1 and 2 years) | SF36 / EuroQoL / GOS  Higher QoL in women with higher increase of all scale at 2 years |
| Llaquet Bayo et al. 2019^68^ | Prospective cohort | 200 | Spain | F | M | Determine whether quality of life is recovered completely after major injury and to identify determinants associated with a worse quality of life | EQ-5D at 1 year  Female gender and age ≥ 55 years are statistically significant determinants of poorer EQvas and EQus. |
| Tan et al. 2018^69^ | Retrospective observational | 478 | Singapore | M | F | to compare the Glasgow Outcome Scale-Extended (GOSE) at 6 months and 12 months in blunt trauma survivors with an Injury Severity Score (ISS) of more than 15 | Females experience worse functional outcomes at 12 months, potentially due to majority of female injuries being low falls in the elderly. In contrast, motor vehicle injury patients had better functional outcomes at 12 months. |
| Ringburg et al. 2010^70^ | Prospective cohort | 246 | Netherlands | M | F | Assess the HRQoL of survivors of severe trauma 1 year after injury, specified according to all the separate dimensions of the EQ-5D and the HUI. | No return to normal QoL at 1 year. Female gender and comorbidity are predictors of long-term disability. |
| Lotfalla et al. 2023^71^ | Systematic review | - | Netherlands | F | M | Assess which factors affect the health-related quality of life | Female gender was a predictor for worse health related quality of life |
| Mostafa et al. 2002^80^ | Retrospective observational | 612 | USA | M | M | examined genderrelated  outcomes in trauma patients | Female trauma patients had better  Outcomes (lower mortality) than male patients in the younger age group. Outcome in the older age group was not gender-related |
| Magnotti et al. 2008^79^ | Retrospective observational | 36,010 | USA | M | M | evaluate the effect of gender on various outcomes of trauma patients after blunt injury | Gender is not independently associated with mortality after blunt trauma |

ISS: Injury Severity Score; MODS : Multi Organ Dysfunction Syndrome; PCT : Procalcitonin; IL : Interleukine; RR: Respiratory rate; SBP: Systolic blood pressure; RTS: Revised Trauma Score; MOI: Mechanism of Injury; BMI: Body Mass Index; GCS: Glasgow Coma Scale; AIS: Abbreviated Injury Scale; MOF: Multi Organ Failure; ARDS: Acute Respiratory Distress Syndrome; PE: Pulmonary Embolism; DVT: deep Venous Thrombosis; UTI: Urinary Tract Infection; ARF: Acute Renal Failure; LOS: Length of Stay; PTX: Pneumothorax; FAST: Focused Assessment Sonography for Trauma; PSM: Propensity Score Matching.

sTable 4. Characteristics of the study about traumatic brain injury by gender.

| Author | Study design | N | Country | Sex of authors  First / Last | | Main objective of the study | outcome |
| --- | --- | --- | --- | --- | --- | --- | --- |
| Kraus et al, 2000^76^ | Prospective cohort | 795 | USA | M | M | Role of gender as factor in outcomes following moderate or severe TBI (adjusted for penetrating, multiple trauma, GCS categories, age categories) | In-hospital death (1H, 1-6H, at discharge, at 18 months) /  Women were 1.57 more likely to have poor outcomes |
| Davis et al, 2006^74^ | Retrospective cohort | 13,437 | USA | M | M | Relationship between gender and outcome in moderate to severe TBI (stratification by pre and post-menopausal age and adjusted for TRISS) | Survival at hospital discharge /  No difference in premenopausal female. Lower mortality in older women (no neuroprotective effect). |
| Ng et al, 2006^75^ | Prospective cohort | 672 | Singapore | M | M | Sex-based difference in outcome at 6 months in TBI admitted in ICU (adjusted for age, multiple injuries, GCS, pupil) | GOS at 6 months /  Higher mortality and poor outcome in women independently of pre and post-menopausal age. |
| Yeung et al, 2011^73^ | Retrospective cohort | 2,979 | Hong Kong, Australia | F | M | Association between sex and mortality in TBI in patients aged 12 to 45 y (adjusted for isolated TBI, multiple trauma, comorbidities, SBP, transfer, RR, GCS, ICU admission, Head surgery, ISS, SDH, EPH, SAH, Hematoma) | In-hospital death /  No difference in mortality |
| Albrecht et al, 2016^72^ | Retrospective cohort | 4,854 | USA | F | M | Assess in-hospital mortality in older adults with TBI by gender (adjusted for AIS, GCS, race, SBP, transfer to TC) | In-hospital mortality /  No difference in mortality in isolated TBI |
| Mair et al. 2022^77^ | Retrospective cohort | 42,034 | Germany | F | M | Assess gender-dependent differences in mortality after TBI. | Matched pair analysis.  Better outcome for women 29.2% vs 30.8% (however unadjusted analysis and expected mortality based on RISC II was 1.4% lower…) |

TBI: Traumatic Brain Injury; GCS: Glasgow Coma Scale; TRISS : Trauma Score and Injury Severity Score; ICU : Intensive Care Unit; GOS : Glasgow Outcome Scale; SBP : Systolic Blood Pressure; RR: Respiratory Rate; ISS: Injury Severity Score; SDH: Subdural hematoma; EPH: Epidural Hematoma; SAH: Subarachnoid haemorrhage; AIS; Abbreviated Injury Scale; TC: Trauma Centre; MOI: Mechanism of Injury.
